# A dramatic, objective antiandrogen withdrawal response: case report and review of the literature

**DOI:** 10.1186/1756-8722-1-21

**Published:** 2008-11-05

**Authors:** Yiu-Keung Lau, Manpreet K Chadha, Alan Litwin, Donald L Trump

**Affiliations:** 1 Hematology/Oncology, Department of Medicine, West Virginia University, West Virginia, USA; 2 Department of Medicine, Roswell Park Cancer Institute, Elm and Carlton Streets, Buffalo, NY, USA; 3 Department of Radiology, Roswell Park Cancer Institute, Elm and Carlton Streets, Buffalo, NY, USA

## Abstract

Antiandrogen withdrawal response is an increasingly recognized entity in patients with metastatic prostate cancer. To our knowledge, there have been no reports describing a *durable *radiologic improvement along with prostate-specific antigen (PSA) with discontinuation of the antiandrogen agent bicalutamide. We report a case in which a dramatic decline of serum PSA levels associated with a dramatic improvement in radiologic disease was achieved with bicalutamide discontinuation.

## Background

Cancer of the prostate is the most prevalent cancer of American men [1]. At the time of diagnosis almost 50% of the patients have disease that extends beyond the prostate gland. Disseminated prostate cancer is primarily treated by local palliative measures and by testicular androgen ablation (medical or surgical). Non-steroidal antiandrogens are commonly used – either as short-term induction therapy to blunt the surge of testosterone that follows the initiation of luteinizing hormone-releasing hormone (LHRH) analogues, as long-term therapy with LHRH analogues or as single agent salvage treatment in men in whom LHRH analogues or surgical castration have ceased to control the disease. A phenomenon referred to as the antiandrogen withdrawal syndrome or antiandrogen withdrawal response (AAWR) occurring in men receiving non-steroidal antiandrogens was first described in 1993 [2-4]. The AAWR is defined as a 50% decline in prostate specific antigen (PSA) following cessation of an antiandrogen. The pathophysiology of the phenomenon is not completely understood. We report a very dramatic and prolonged antiandrogen withdrawal response and discuss the literature and recent information regarding the pathophysiology of the AAWR.

## Case presentation

**Figure 1:**
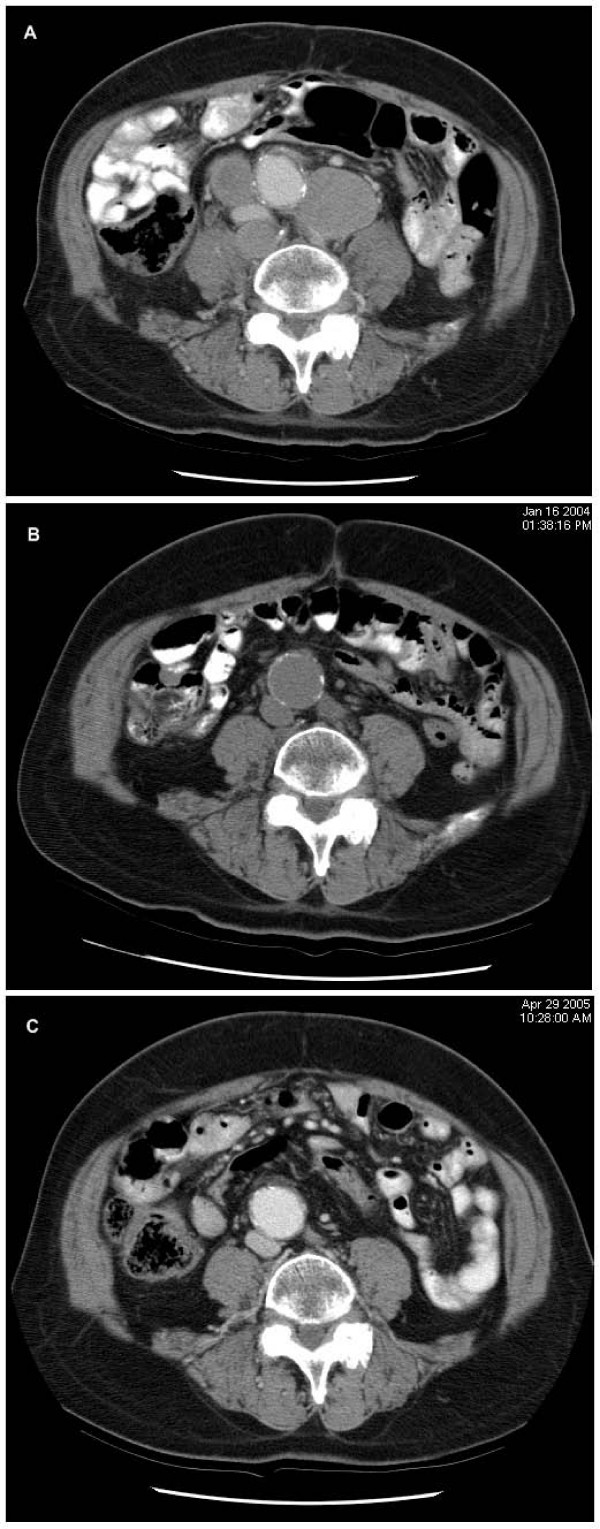
**Radiographic response to Bicalutamide withdrawal.** Panel A depicts computed tomography scan of abdomen and pelvis with significant periaortic and retroperitoneal adenopathy about 2 months after cessation of bicalutamide. Panel B depicts dramatic egression of adenopathy in response to bicalutamide withdrawal after 8 months. Panel C depicts durable response 20 months after bicalutamide withdrawal.

**Figure 2:**
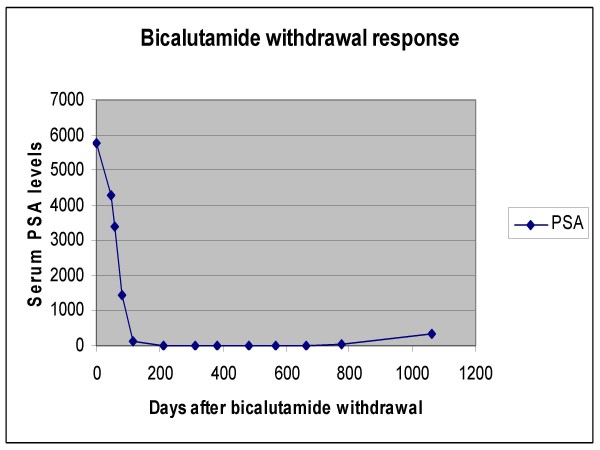
**Effect of cessation of bicalutamide on serum PSA concentrations.** Day zero is day of bicalutamide discontinutation.

The patient is a 75-year old African American man in whom prostate adenocarcinoma was initially diagnosed in 1996. He had been asymptomatic and had presented with an increased prostate specific antigen (PSA) and an enlarged prostate on physical exam. Biopsy of the prostate revealed prostate adenocarcinoma, Gleason grade 4+5 = 9/10. His PSA at the time of diagnosis was 3105 ng/mL. A bone scan showed no evidence of metastatic disease, and there are no records of other imaging studies being done. He was treated first with complete androgen blockade (leuprolide every three months and bicalutamide). The PSA decreased dramatically to 0.55 ng/mL in six months. On follow-up examination, there was shrinkage of the prostate gland. He then received external beam radiation to the prostate gland and the pelvis with total of 5000 cGy from June of 1997 to July of 1997. The post-treatment PSA was less than 0.5 ng/mL. He received brachytherapy with palladium ^103 ^(69 seeds, 1.36 mCi/seed) in August of 1997. His PSA, however, progressively rose: 3.9 ng/mL in 1998, 23.7 ng/mL in 1999, 81.6 ng/mL in 2000 and 144.7 ng/mL in 2001. At that time, he was found to have bladder outlet obstruction, thought to be due to bladder calculi. He underwent lithotripsy for bladder stones and transurethral resection of the prostate. Neither slides nor tissue from this procedure are presently available. Bone scan in June of 2001 did not show bone metastases. The serum PSA level rose to 4944 ng/mL in June of 2003. He had been maintained on leuprolide and bicalutamide since 1996. He was first seen at Roswell Park in June 2003. PSA level was 5786 ng/mL. He was asymptomatic and physical examination revealed residual mild expressive aphasia and mild weakness in the left upper extremity due to a remote cerebrovascular accident. His performance status was Eastern Cooperative Oncology Group score of 0–1 and there were no other significant laboratory abnormalities except for mildly elevated serum creatinine. The prostate was not enlarged on physical exam. Anticipating the possibility of an AAWR, we recommended discontinuation of bilcalutmide. The PSA declined by 26% (from 5789 to 4275 ng/mL) after 45 days of antiandrogen withdrawal. In the meantime, we obtained imaging studies to evaluate his disease. Abdominal and pelvic CT scans showed massive retroperitoneal, bilateral common iliac and bilateral internal iliac adenopathy consistent with metastatic disease (see Figure 1A). Bone scan did not show any evidence of bone metastases. The serum PSA concentration continued to decrease and two months following discontinuation of bicalutamide it was 3376 ng/mL (45% decline). Twelve months after discontinuing bicalutamide the PSA reached a nadir of 3.19 ng/mL (Figure 2). Repeat abdominal and pelvic CT scan 7 months after the discontinuation of bicalutamide showed remarkable reduction in retroperitoneal and iliac adenopathy (Figure 1B). The patient continued to do well clinically. Twenty months after anti-androgen withdrawal, the radiological response was maintained on the abdominal and pelvic CT scan (Figure 1C). Patient remained on leuprolide alone (after biacalutamide withdrawal) for approximately 3 years till significant increase in PSA was noted and therapy changed to ketoconazole and hydrocortisone in addition to leuprolide. At the time of change of therapy (May 2006) PSA had increased to 331 ng/ml. No significant change in the retroperitoneal adenopathy was noted at that time on CT imaging. Interval development of nodularity adjacent to and contiguous with the superior aspect of the prostate gland and a lymph node in the right obturator region were noted, which were new. Patient was asymptomatic. Patient was on multiple medications which interacted with ketoconazole and patient chose to continue on hydrocortisone alone after a short period (less than a month). PSA declined to 222.70 ng/ml in June 2006 but increased thereafter to 724.89 ng/ml in December 2006. Performance status continued to decline and PSA progressed rapidly to 4008.17 ng/ml in March 2007 and to 5888.5 ng/ml in April 2007. Patient at this time decided to restart ketoconazole in addition to hydrocortisone. However overall condition declined rapidly and patient was placed under hospice care and died in September 2007

## Discussion

### Prior case reports and clinical studies in antiandrogen withdrawal

Table 1 summarizes a series of reports on antiandrogen withdrawal [5-19].

**Table 1:** Reports of antiandrogen withdrawal

**Author**	**Antiandrogen agent(s)**	**No. of Patients**	**No. of patients with 50% PSA decline**	**Duration of Anti-androgen therapy (Months)**	**Duration of AAWR (Months)**
Kelly[5]	Flutamide	3	2	5–17	2–5
Collinson[17]	Flutamide	1	1	12	12
Scher[6]	Flutamide	36	10	NS	5*
Scher[15]	Flutamide	57	16	16	5.6
Dupont[7]	Flutamide†	40	32	5–7	14.5
Figg[16]	Flutamide	21	7	28	3.7
Sartor[18]	Flutamide†	29	14	Median: 27*	8
Herrade[8]	Flutamide	39	11	Median: 19*	3.3
Small[9]	Flutamide	82	12	Median: 21.5*	3.5
Small[10]	Bicalutamide	1	1	32	2
Nieh[19]	Bicalutamide	3	2	24–35	2–4
Schellhammer [11]	Flutamide/Bicalutamide‡	22	8	16 months combined with LHRH agonist	NS
Small[14]	Flutamide/Bicalutamide/Nilutamide	132	15	NS	5.9
Gomella[12]	Nilutamide	1	1	11	3
Huan[13]	Nilutamide††	2	2	10 and 15	7 and 6

NS = not stated

* indicates median

† Adjuvant treatment with aminoglutamide and hydrocortisone

‡ 4 patients assessed for objective response at 12 weeks: 2 has stable disease

†† One patient was treated with flutamide, and then cyproterone before nilutamide; Another patient were treated with combined goserelin and nilutamide

The AAWR was first described by Kelly and Scher in 1993 [5]. They described three metastatic prostate cancer patients in whom discontinuation of the flutamide resulted in PSA decrease (36% to 89% decline) and in some cases symptomatic improvement. In a second report by the same group, 36 men with metastatic prostate cancer who were receiving flutamide and had progressive disease were evaluated following flutamide discontinuation [6]. Ten patients had a substantial decline (≥ 80% in 7 patients and ≥ 50% in 3) in the serum PSA level. The duration of decline was short, median of 5+ months. In a Canadian study, the median duration of response was 14.5 months [7]. Other reports of flutamide withdrawal response are as noted in table 1[8,9]. AAWR has also been described following bicalutamide and nilutamide withdrawal [10-13]. Small and colleagues compared antiandrogen withdrawal alone or in combination with ketoconazole in a randomized phase III trial in androgen-independent prostate cancer patients [14]. This is the largest prospective study of AAWR. One hundred thirty-two patients were randomized to undergo androgen withdrawal alone or with ketoconazole and hydrocortisone. Eleven percent of patients undergoing antiandrogen withdrawal alone experienced PSA decline = 50%. The objective response rate in a measurable disease was 2%. The median time to PSA progression in PSA responders was 5.9 months (5.3 to 10.1 months). Of the patients had antiandrogen withdrawal and ketoconazole, 27% had a PSA response and 20% had objective responses. There were no differences in survival.

Sher and colleagues suggested that there was an association between duration of antiandrogen therapy and the likelihood of AAWR [15]. The median duration of antiandrogen use for patients with a PSA decline greater than 50% from the baseline was 25 months versus 16 months of those nonresponders (p value = 0.012). Figg et al reported that patients who responded to flutamide withdrawal received the agent for a longer period (2.33 years) than the non-responders (1.54 years) [16]. These differences were not statistically significant. The study from Herrade et al did not show the significant association between the duration of its use and withdrawal response [8]. The median months for initial hormone therapy were 19 months for the responders and 16.5 months for the non-responders (p = 0.41). There are no clear "predictors" of AAWR.

### Molecular mechanisms of antiandrogen withdrawal response

**Figure 3:**
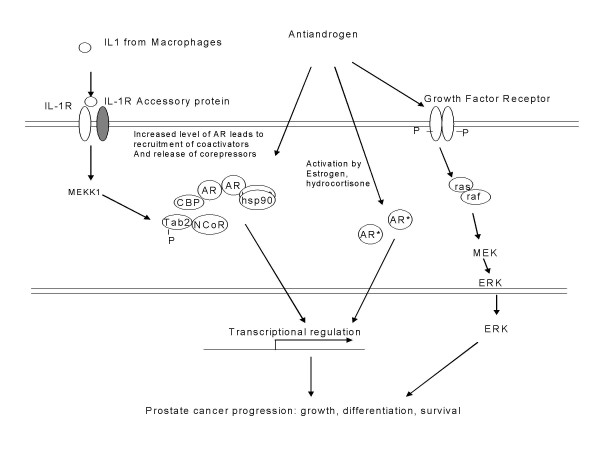
**Schematic diagram for different proposed mechanisms of antiandrogen withdrawal.***AR*, androgen receptor, AR* mutate androgen receptor, IL-1R, interleukin-1 receptor, hsp90, heat shock protein 90, MEKK, mitogen-activated protein/ERK kinase kinase.

The mechanisms of AAWR are also unclear. Several proposed mechanisms are depicted in Figure 3[20-28]. The androgen receptor (AR) belongs to the steroid-receptor superfamily. After binding to its ligand, androgen, AR forms a homodimer and regulates androgen responsive genes via androgen response elements. The receptor functions as a transcriptional factor and together with other co-regulatory proteins (coactivators and co-repressors) which may associate with the AR-ligand complex control transcriptional activity. AR consists of a c-terminal ligand binding domain and a transactivation domain, the central DNA binding domain and the N-terminal transactivation domain. The AR protein can be detected even in the androgen-independent prostate cancer and AR signaling is believed to be important even in so-called "androgen independent disease". One of the proposed mechanisms of the AAWR is that it is mediated through AR mutation. It is postulated that mutations in the ligand binding domain can render noncanonical ligands, such as estrogen, hydrocortisone, or androgen receptor antagonists, agnostic[22,23]. *In vitro*, hydroxyflutamide activates AR transcriptional activity in the androgen-sensitive prostate cancer cell line, LNCaP. In LNCaP there is a point mutation in the AR, (threonine to alanine), at codon 877 in the ligand binding domain of the *AR *gene [20,22]. Such mutations have been described in tumor cells from patients who showed the AAWR [21]. Taplin et al have described the isolation of cancer cells with mutant androgen receptor genes from patients with metastatic, androgen-independent cancer patients [24]. When these cloned genes were transfected into the CV-1 cells (monkey fibroblast cell line), they responded in an agonistic fashion to progesterone and estradiol. The wild type androgen receptor is unresponsive or only weakly activated by antiandrogens. However, a detailed study by this group showed that only 10% of surveyed samples of tumor tissue from patients with androgen independent disease demonstrated AR mutations[25]. One hundred eighty-four bone marrow biopsies were obtained from men enrolled on a trial of antiandrogen withdrawal. In 48 bone marrow biopsies prostate cancer was detected. The androgen receptors from these samples were sequenced. Five out of 48 samples (10%) had mutated androgen receptors. All mutations were single-base, missense substitutions. Two of the mutations were located in the AF-2 domain of androgen receptor which is essential for coactivator binding and its signaling. They found no correlation between androgen receptor mutations and an antiandrogen withdrawal response or survival. There was a 20% PSA response to antiandrogen withdrawal in the androgen receptor mutations group, compared with a 7% PSA response in the no mutation group. The median survival in the androgen receptor mutation group was 9.2 months, compared with 11.2 months in the no mutation group. The progression-free survival was 3.3 months in the AR mutation group, compared with 3.1 months in the no mutation group. Therefore, mutations alone seem unlikely to account for all cases of androgen independent disease and the antiandrogen withdrawal response.

It has also been suggested that antiandrogens may stimulate a non-androgen receptor pathway. Lee et al showed that hydroxyflutamide can activate the mitogen-activated protein (MAP) kinase pathway independent of androgen receptor [26]. They showed by immunohistochemical analysis a significant increase of activated MAP kinase in prostate tumors from patients receiving hydroxyflutamide. *In vitro *hydroxyflutmamide induced rapid activation of ras/MAP kinase pathway in human prostate cancer DU145 cells (which lack the androgen receptor) as well as in CWR22 and PC-3AR2 androgen receptor positive cells lines, indicating that the activation did not require androgen receptor. Furthermore, an epidermal growth factor receptor inhibitor or neutralizing antibody could obliterate this hydroxyflutamine-mediated activation of MAP kinase pathway. They suggested that antiandrogen, hydroxyflutamide could initiate the activation via a membrane-initiated, non-androgen receptor-mediated action, providing alternative pathway that might contribute to the withdrawal syndrome. However, there is no report of other nonsteroidal antiandrogen, bicalutamide and nilutamide, having such stimulatory effect on the MAP kinase pathway, limiting the generalizability of this finding.

Another mechanism could be over-expression of androgen receptor. Taplin et al showed that all metastatic androgen-independent tumors examined in her study expressed high levels of androgen receptor gene transcripts, relative to the levels expressed by an androgen dependent prostate cancer cell line, LNCaP [24]. This raises the possibility of increasing androgen receptor expression may alter the prostate cancer cells' response to its ligand or even its antagonists. A report by Chen et al showed that mere 3-fold increase in androgen receptor will confer prostate cancer cells resistance to antiandrogens by amplifying signal output from low levels of residual ligand, and by altering the normal response to antagonists [27]. The study showed that when androgen receptors were over-expressed by transfection in the prostate cancer cell lines, LNCaP and LAPC4, the transfected cells were able to grow in low androgen concentrations. Importantly, the cell growth could also be enhanced by the antiandrogen, bicalutamide. The possible explanation could be an aberrant interaction between an androgen receptor coregulatory proteins and the receptor itself. They found that high androgen receptor levels could alter coactivator assembly with subsequent effects on transcriptional activity.

Most recently, studies of macrophage/cancer cell interaction have provided another possible explanation for hormone resistance in the androgen-independent prostate cancer and the AAWR [28]. Zhu et al noted that all tumor samples they examined exhibited macrophage infiltration as well as stromal interactions with macrophages as compared to much less interaction between the macrophage and the normal areas [28]. The findings suggest modulatory effects of macrophages on the cancer cells through the cytokines such as interleukin-1. They subsequently showed that IL-1 could convert androgen antagonists into agonists. The IL-1 stimulation of mitogen-activated protein/ERK kinase kinases (MEKK) results in phosphorylation of TAB2, an AR-interacting protein, which resulted in and release of the TAB2/N-CoR holocorepressor complex from the androgen receptor. Transcriptional activity of the androgen receptor, which was inhibited in the presence of bicalutamide, was then turned on.

## Conclusion

In conclusion, we report here one of the first and to our knowledge most dramatic, and sustained antiandrogen withdrawal response. This case report emphasizes this subgroup of patients may have significant response to antiandrogen withdrawal and benefit from a non-cytotoxic intervention. Identification of this group of patients is important. Even though the exact mechanism of the antiandrogen withdrawal is not known, the molecular data suggest that androgen receptor still plays a crucial role in the phenomenon. Further understanding of the receptor biology is important.

## Abbreviations

AAWR: anti-androgen withdrawal; AR: androgen receptor; CT: computed tomography; PSA: prostate specific antigen.

## Consent

Written informed consent could not be obtained in this case since the patient is deceased and the next of kin were untraceable. The Editors believe this case report contains a worthwhile clinical lesson, which could not be as effectively made in any other way. The Editors also expect the next of kin (or reasonable person) not to object to the publication since details of the patient remains anonymous.

## Competing interests

The authors declare that they have no competing interests.

## Authors' contributions

All authors were involved in preparation of this manuscript, including data collection and preparation of figures.
